# Disseminated Tuberculosis With Pericardial Effusion and Early Tamponade: A Case Report

**DOI:** 10.7759/cureus.82632

**Published:** 2025-04-20

**Authors:** Usamah Al-Anbagi, Ali A Alkeldi, Claret Charles Isabirye, Abdulqadir J Nashwan

**Affiliations:** 1 Internal Medicine Department, Hazm Mebaireek General Hospital/Hamad Medical Corporation, Doha, QAT; 2 Medical Education Department, Hamad Medical Corporation, Doha, QAT; 3 Internal Medicine Department, Hamad Medical Corporation, Doha, QAT; 4 Nursing & Midwifery Research Department, Hamad Medical Corporation, Doha, QAT

**Keywords:** anti tuberculosis therapy, cardiac tamponade, mycobacterium tuberculosis (mtb), pericardial effusion, pericardiocentesis, pericarditis, steroid

## Abstract

Tuberculous pericarditis is a rare but life-threatening complication of tuberculosis (TB), often presenting with nonspecific symptoms and leading to delayed diagnosis. This case report describes a 26-year-old male with disseminated TB involving the pericardium, pleura, and mediastinal lymph nodes, complicated by early cardiac tamponade. Despite the presence of tamponade physiology, the patient was successfully managed with anti-TB therapy and corticosteroids without invasive intervention. Diagnostic challenges, including the role of imaging, fluid analysis, and molecular testing, are discussed. The case highlights the importance of early diagnosis, timely initiation of anti-TB therapy, and the potential for non-interventional management in select cases. Clinical improvement and resolution of tamponade physiology on follow-up echocardiography underscore the efficacy of medical therapy in preventing complications such as constrictive pericarditis. This report emphasizes the need for a high index of suspicion in TB-endemic regions and the value of individualized treatment approaches.

## Introduction

Tuberculous pericarditis is a significant extrapulmonary manifestation of TB, accounting for approximately 1-2% of pulmonary TB cases [1]. It arises from the spread of *Mycobacterium tuberculosis* to the pericardium, either through direct extension from adjacent structures or hematogenous dissemination during miliary TB [1]. Despite advances in TB management, tuberculous pericarditis remains a diagnostic and therapeutic challenge, often presenting with nonspecific symptoms, such as fever, weight loss, and night sweats, which can delay diagnosis and increase the risk of complications like constrictive pericarditis and cardiac tamponade [2].

The disease progresses through four pathological stages: fibrinous exudation, serosanguineous effusion, absorption with fibrosis, and constrictive scarring [3]. Without timely treatment, constrictive pericarditis develops in 30-60% of cases, significantly increasing morbidity and mortality [3]. Diagnostic confirmation relies on detecting M. tuberculosis in pericardial fluid or tissue, though presumptive diagnosis is often based on clinical, radiological, and biochemical findings, particularly in TB-endemic regions [4].

This case report describes a 26-year-old male with disseminated TB involving the pericardium, pleura, and mediastinal lymph nodes, complicated by early cardiac tamponade. The patient was successfully managed with anti-TB therapy and corticosteroids without invasive intervention, demonstrating the potential for non-interventional management in select cases. The report underscores the importance of early diagnosis, timely treatment, and individualized approaches to prevent life-threatening complications.

## Case presentation

A 26-year-old male with no significant past medical history presented to the Emergency Department with a three-week history of dry cough, worsening at night, and no associated hemoptysis. He also reported past exertional dyspnea, loss of appetite, weight loss of 1 kg over the past two weeks, and night sweats. Over the preceding two weeks, he developed intermittent fever, chills, fatigue, left-sided pleuritic chest pain, and a few episodes of vomiting. There was no history of orthopnea, paroxysmal nocturnal dyspnea, palpitations, dizziness, or syncope. He denied recent travel, sick contacts, or TB exposure. He lives with seven asymptomatic individuals and works as a security guard. He is a non-smoker, non-alcoholic, and has no known drug allergies.

Examination

On examination, the patient was clinically stable and comfortable. Vital signs included a temperature of 36.8 °C, heart rate of 100 bpm, respiratory rate of 18 breaths/min, blood pressure of 123/84 mmHg, and oxygen saturation (SpO2) of 99% on room air. A soft, <0.5 cm, left lower anterior cervical lymph node was palpable. A cardiovascular examination revealed normal heart sounds (S1, S2), a positive pericardial rub, and no murmurs or peripheral edema. Respiratory examination demonstrated bilateral lower zone crepitations and dullness on percussion in the infra-scapular and axillary regions. The abdominal examination was unremarkable, with no tenderness, guarding, or organomegaly. Neurological examination revealed a Glasgow Coma Score (GCS) of 15/15 with no focal deficits.

Investigations

Initial laboratory investigations revealed mild anemia, elevated C-reactive protein (CRP), prolonged prothrombin time (PT) and international normalized ratio (INR), with normal white blood cell count (WBC) and procalcitonin levels (Table 1).

**Table 1:** Laboratory Investigations Total Leukocytes (WBC), Hematocrit (HCT), Hemoglobin (HGB), Mean Corpuscular Volume (MCV), Mean Corpuscular Hemoglobin (MCH), Platelet Count (PLT), Serum Potassium (K), Serum Sodium (Na), Serum Urea (Urea), Serum Creatinine (Cr), Serum Albumin (ALB), Serum Total Protein (TP), Aspartate Aminotransferase (AST), Alanine Aminotransferase (ALT), Alkaline Phosphatase (ALP), Serum Total Bilirubin (TBIL), C-Reactive Protein (CRP), Human Immunodeficiency Virus (HIV)

Parameters	On admission	^3rd^ day	On discharge	Reference values
Total leukocytes	4.5	4.4	7.8	(6.2 x10^3/uL)
Hematocrit	33	37	35.6	(40-50%)
Hemoglobin (gm/dL)	10.7	12.2	11.6	(13-17 gm/dL)
MCV (fL)	83	81	81	(83-101 fL)
MCH (pg)	26.9	26.5	26.6	(27-32 pg)
Platelet (x10^3/uL)	393	443	435	(150-410 x10^3/uL)
Serum potassium K (mmol/L)	4.7	4.8	4.2	(3.5-5.3)
Serum sodium (mmol/L)	133	137	137	(133-146)
Serum urea (mmol/L)	201	3.8	4.9	(2.5-7.8)
Serum creatinine (umol/L)	65	62	62	(62-106)
Serum albumin (gm/L)	28	28	29	(35-50)
Serum total protein (gm/L)	71	76	74	(60-80)
AST (IU/L)	36	58	22	(0-41)
ALT (IU/L)	45	66	49	(0-41)
Alkaline phosphatase (U/L)	162	184	170	(40–129)
Serum total bilirubin (mg/dl)	10	6	4	(0-21)
CRP (mg/L)	100	108	128	(0-5 mg/L)
HIV	-	Negative	-	Negative

**Figure 1:**
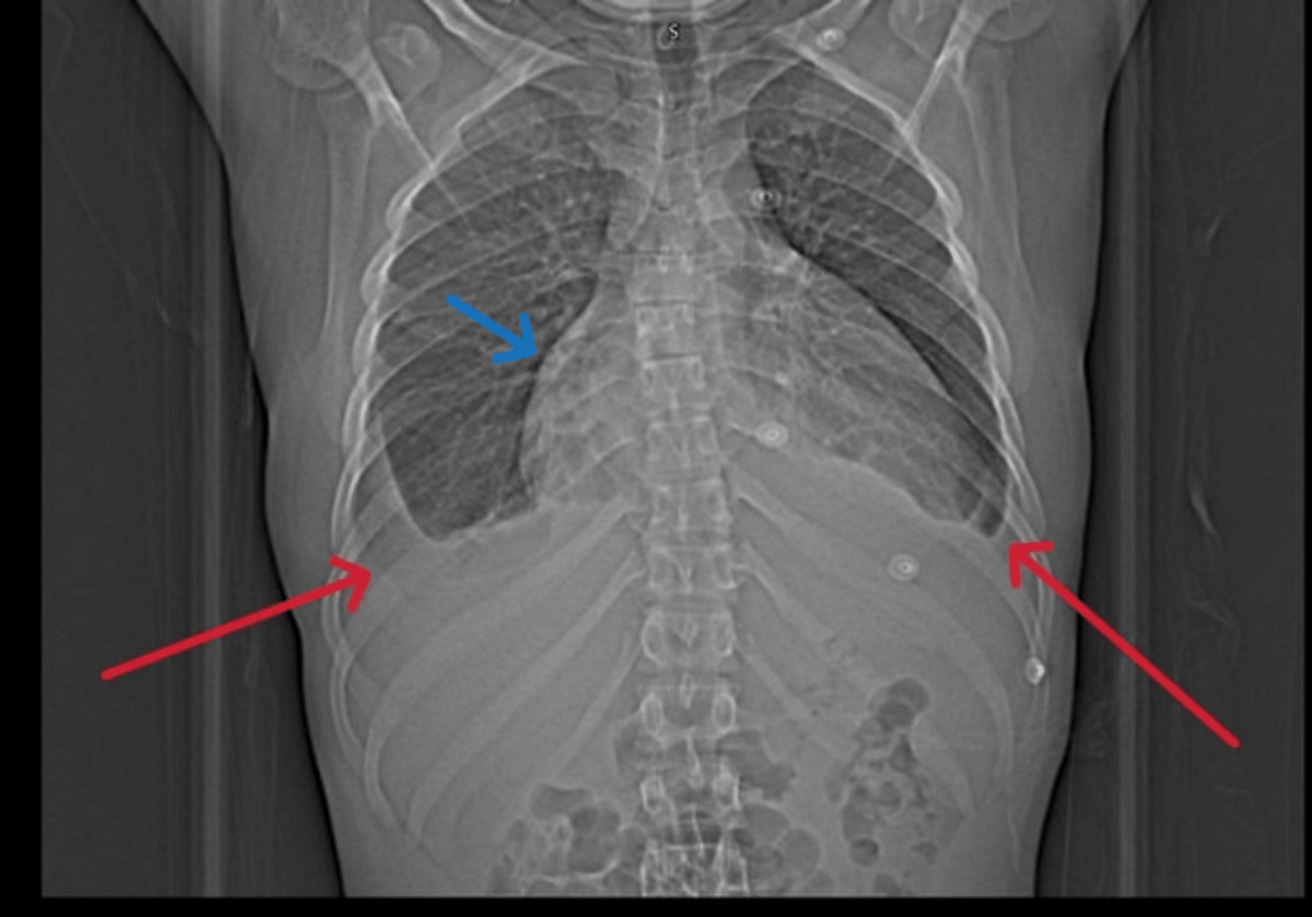
Chest X-ray (PA view) The red arrows showed bilateral pleural effusion, the blue arrow revealed cardiomegaly with pericardial effusion. PA: posteroanterior

**Figure 2:**
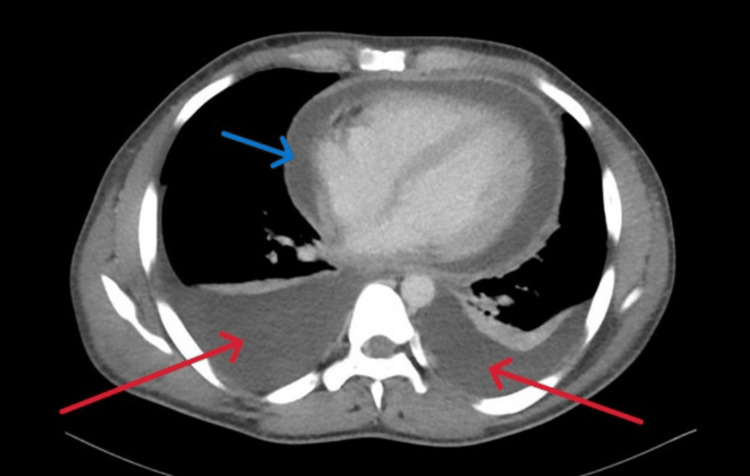
Axial CT thorax The red arrows showed bilateral pleural effusion, the blue arrow revealed cardiomegaly with pericardial effusion.

Sputum screening for TB was negative. Imaging studies included a chest X-ray (Figure 1), which showed bilateral pleural effusion and cardiomegaly, and CT thorax, which revealed pericarditis with significant pericardial effusion, bilateral pleural effusion, and multiple low-attenuation mediastinal lymph nodes suggestive of TB lymphadenitis (Figure 2).

Initial echocardiography demonstrated a large circumferential pericardial effusion with mild thickening and effusive-constrictive pericarditis but no hemodynamic compromise. Repeated echocardiogram 3 days later confirmed the presence of early cardiac tamponade based on a dilated IVC with <50% inspiratory collapse, large pericardial effusion with evidence of increased intrapericardial pressure (early tamponade physiology), respiratory variation ≥30% in mitral valve inflow and <60% in tricuspid valve inflow, and mild septal bounce. Diagnostic pleural fluid analysis revealed exudative, lymphocytic fluid with positive TB smear and PCR.

Hospital course and management

The patient was initially started on empirical treatment with ceftriaxone (2 g daily for 7 days), colchicine (0.5 mg twice daily), and ibuprofen (400 mg every 8 hours). Cardiology consultation recommended close monitoring with no immediate need for pericardiocentesis, and the patient was transferred to telemetry with a follow-up echocardiography planned. Repeat echocardiography after three days showed worsening pericardial effusion with early cardiac tamponade, prompting transfer to the CCU for consideration of diagnostic/therapeutic pericardiocentesis. Anti-TB treatment was initiated with a fixed-dose combination (rifampicin 150 mg, isoniazid 75 mg, pyrazinamide 400 mg, ethambutol 275 mg) along with pyridoxine 40 mg daily, with a plan for a full course of 9-12 months. Prednisolone 60 mg daily was added on day 5 to manage pericardial inflammation, with a plan for a tapering course over a few weeks. The patient showed significant clinical improvement, and pericardiocentesis was ultimately deferred due to stabilization. He was discharged on day 9 in stable condition with a final diagnosis of disseminated TB infection involving the mediastinum, pericardium, and pleura.

Follow-up

**Figure 3:**
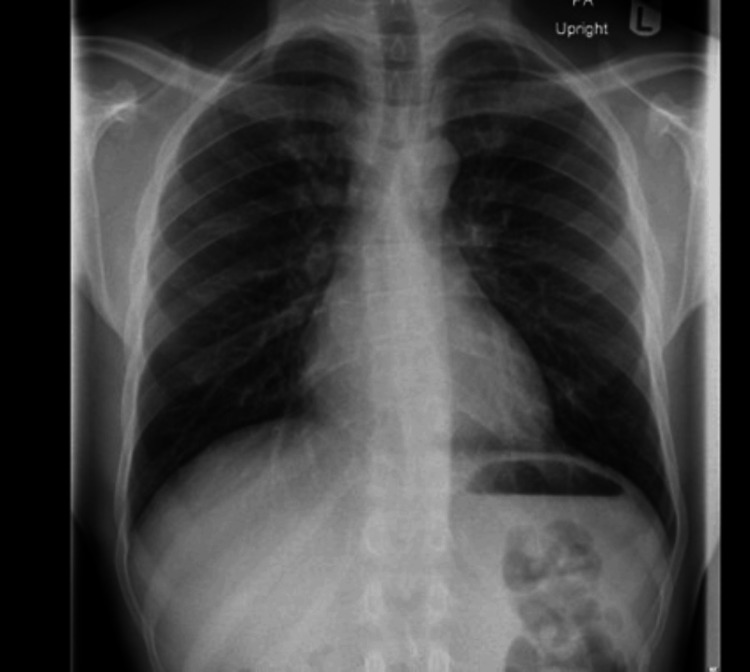
Chest X-ray (PA view) CXR revealed improvement in both pleural and pericardial effusion. PA: posteroanterior

Follow-ups at three days and three weeks post-discharge revealed significant clinical improvement. Repeat echocardiography showed mild residual pericardial effusion, and he remained stable on tapering doses of prednisolone and continued anti-TB therapy. No further complications were reported. A follow-up chest X-ray (CXR) two months later demonstrated significant improvement in pleural and pericardial effusion compared to previous findings (Figure 3).

## Discussion

Tuberculous pericarditis is a significant complication of tuberculosis, often presenting diagnostic challenges that can lead to delayed or missed diagnoses. This delay increases the risk of late complications such as constrictive pericarditis and higher mortality [1]. Tuberculous pericarditis occurs in approximately 1-2% of patients with pulmonary TB [2]. In a series of 294 immunocompetent patients in Spain with acute pericarditis of unknown cause, tuberculous pericarditis was diagnosed in 13 patients (4%) [3], with cardiac tamponade observed in 5 and constrictive pericarditis developing in 6 [3]. In regions with high HIV prevalence, the incidence of tuberculous pericarditis has risen dramatically. For example, a 1994 study in Tanzania found that all HIV-infected patients with large pericardial effusions had tuberculous pericarditis [4].

Tuberculous pericarditis results from Mycobacterium tuberculosis spreading to the pericardium through direct extension from adjacent structures (e.g., lungs, lymph nodes, or bones) or via hematogenous dissemination during miliary TB. It often represents the reactivation of a latent infection, with the primary focus frequently undetectable. The disease progresses through four stages: 1) fibrinous exudation with granuloma formation, 2) serosanguineous effusion with lymphocytic exudate, 3) absorption of effusion with fibrosis, and 4) constrictive scarring, which can lead to calcification and impaired cardiac filling [5-6]. These stages may occur sequentially or independently, with the effusive stage often being the earliest detectable phase, reflecting a hypersensitivity reaction to tubercular antigens. Without treatment, effusions may resolve spontaneously in about 50% of cases within 2-4 weeks, but constriction can develop unpredictably [7]. In some cases, effusive-constrictive pericarditis occurs, characterized by concurrent effusion and constriction, where fibrosis and fluid accumulation complicate management.

Tuberculous pericarditis presents with nonspecific symptoms, such as fever, weight loss, and night sweats, often preceding cardiopulmonary complaints. Common symptoms include cough, dyspnea, pleuritic chest pain, and orthopnea, though their frequency varies [5]. Physical findings may include fever, tachycardia, elevated jugular venous pressure, hepatomegaly, ascites, peripheral edema, and a pericardial friction rub [8-9]. Complications include constrictive pericarditis (30-60% of cases) [4], effusive-constrictive pericarditis (persistent constriction after effusion drainage) [10], and myopericarditis (pericarditis with myocardial involvement, often linked to HIV) [11]. Cardiac tamponade, marked by pulsus paradoxus and hypotension, occurs in about 10% of cases [12]. Constrictive pericarditis may present with Kussmaul’s sign and elevated jugular veins, while effusive-constrictive pericarditis is challenging to diagnose and is often identified during pericardiocentesis. Suspect tuberculous pericarditis in patients with pericarditis and TB risk factors, especially if symptoms persist [3].

Diagnosis is confirmed by detecting M. tuberculosis in pericardial fluid (smear/culture) or caseating granulomas on histology [6]. Presumptive diagnosis is supported by TB elsewhere, lymphocytic exudate with elevated ADA, or response to anti-tuberculous therapy. Initial evaluation includes chest radiography (cardiomegaly, pleural effusions), echocardiography (effusion/tamponade), and sputum AFB analysis. Computed tomography (CT)/magnetic resonance imaging (MRI) may show pericardial thickening, effusion, or lymphadenopathy. Pericardiocentesis is key for fluid analysis (exudative, lymphocytic predominance) [7]; AFB smears are positive in 40-60% of cases, with higher yields from culture [7,9]. Polymerase chain reaction (PCR) for mycobacterial deoxyribonucleic acid (DNA) is also useful in diagnosis; however, the utility for diagnosing extrapulmonary TB is supported by small studies in endemic areas, but its effectiveness in nonendemic regions remains understudied. Measurement of pericardial adenosine deaminase (ADA) levels (cutoff 30-60 units/L) aids diagnosis, though ADA sensitivity may be lower in HIV patients [13]. A pericardial biopsy (granulomas in ~53% of cases) is useful if fluid analysis is inconclusive [14].

Anti-tuberculous therapy has dramatically improved outcomes in tuberculous pericarditis, reducing mortality from 80-90% to 8-17% in HIV-negative patients and 17-34% in HIV-positive patients [15-16]. It also decreases the risk of constrictive pericarditis from 88% to 10-20% in treated cases [17]. The treatment regimen for tuberculous pericarditis is similar to that for pulmonary tuberculosis [18], with adjustments based on HIV status and drug resistance. In TB-endemic areas or when clinical suspicion is high, empiric anti-tuberculous therapy is recommended even before a definitive diagnosis is confirmed, as clinical improvement can support the diagnosis. However, empiric therapy should generally be avoided in nonendemic areas unless there is strong clinical suspicion and no definitive diagnostic evidence. In endemic areas, empiric therapy may be initiated without biopsy if clinical suspicion is high [6].

In this case, a 26-year-old male with disseminated TB involving the pericardium, pleura, and mediastinal lymph nodes presented with early cardiac tamponade. Despite the presence of tamponade physiology, the patient was successfully managed with anti-TB therapy and corticosteroids without invasive intervention. This highlights the importance of early diagnosis, timely initiation of anti-TB treatment, and the potential for non-interventional management in select cases. The patient’s clinical improvement and resolution of tamponade physiology on follow-up echocardiography underscore the efficacy of medical therapy in preventing complications such as constrictive pericarditis.

Corticosteroids are recommended for constrictive tuberculous pericarditis or high-risk cases but show no benefit in non-constrictive cases, especially in HIV patients [19-20]. A 2014 trial in South Africa found no overall benefit in reducing death, tamponade, or constriction, though corticosteroids lowered constriction risk (4.4% vs. 7.8%) but increased HIV-related malignancies [21]. Corticosteroids may hasten symptom resolution and reduce the need for pericardiectomy in constrictive cases, with dosing typically starting at 60 mg/day prednisone, tapered over weeks [22]. A pericardiectomy is indicated for persistent constrictive pericarditis despite anti-tuberculous therapy, though the timing is debated; some advocate early surgery, while others reserve it for non-responders. Surgery is recommended if hemodynamics fail to improve or worsen after 4-8 weeks of therapy, with earlier intervention for pericardial calcification, a sign of chronic disease [23].

## Conclusions

This case highlights the diagnostic and management challenges of disseminated tuberculosis, particularly when complicated by pericardial effusion and early tamponade. The timely initiation of anti-TB therapy, combined with corticosteroids for pericardial inflammation, led to significant clinical improvement without the need for invasive interventions such as pericardiocentesis. The resolution of tamponade physiology on follow-up echocardiography underscores the efficacy of medical management in such cases. This report emphasizes the importance of early diagnosis, individualized treatment approaches, and a high index of suspicion for tuberculous pericarditis, especially in TB-endemic regions.
